# An Updated Global Species Diversity and Phylogeny in the Genus *Wickerhamomyces* with Addition of Two New Species from Thailand

**DOI:** 10.3390/jof7110957

**Published:** 2021-11-11

**Authors:** Supakorn Nundaeng, Nakarin Suwannarach, Savitree Limtong, Surapong Khuna, Jaturong Kumla, Saisamorn Lumyong

**Affiliations:** 1 Master of Science Program in Applied Microbiology (International Program), Faculty of Science, Chiang Mai University, Chiang Mai 50200, Thailand; Supakorn.ning@gmail.com; 2 Department of Biology, Faculty of Science, Chiang Mai University, Chiang Mai 50200, Thailand; suwan.462@gmail.com (N.S.); Trio_9@hotmail.com (S.K.); 3 Research Center of Microbial Diversity and Sustainable Utilization, Chiang Mai University, Chiang Mai 50200, Thailand; 4 Department of Microbiology, Faculty of Science, Kasetsart University, Bangkok 10900, Thailand; fscistl@ku.ac.th; 5 Academy of Science, The Royal Society of Thailand, Bangkok 10300, Thailand

**Keywords:** ascomycetous yeast, distribution, new species, phylogeny, taxonomy, *Wickerhamomyces*

## Abstract

Ascomycetous yeast species in the genus *Wickerhamomyces* (Saccharomycetales, Wickerhamomycetaceae) are isolated from various habitats and distributed throughout the world. Prior to this study, 35 species had been validly published and accepted into this genus. Beneficially, *Wickerhamomyces* species have been used in a number of biotechnologically applications of environment, food, beverage industries, biofuel, medicine and agriculture. However, in some studies, *Wickerhamomyces* species have been identified as an opportunistic human pathogen. Through an overview of diversity, taxonomy and recently published literature, we have updated a brief review of *Wickerhamomyces*. Moreover, two new *Wickerhamomyces* species were isolated from the soil samples of Assam tea (*Camellia sinensis* var. *assamica*) that were collected from plantations in northern Thailand. Herein, we have identified these species as *W*. *lannaensis* and *W*. *nanensis*. The identification of these species was based on phenotypic (morphological, biochemical and physiological characteristics) and molecular analyses. Phylogenetic analyses of a combination of the internal transcribed spacer (ITS) region and the D1/D2 domains of the large subunit (LSU) of ribosomal DNA genes support that *W*. *lannaensis* and *W*. *nanensis* are distinct from other species within the genus *Wickerhamomyces*. A full description, illustrations and a phylogenetic tree showing the position of both new species have been provided. Accordingly, a new combination species, *W*. *myanmarensis* has been proposed based on the phylogenetic results. A new key for species identification is provided.

## 1. Introduction

The genus *Wickerhamomyces* was first proposed by Kurtzman et al. [1] in 2008 with *W*. *canadensis* (basionym *Hansenula canadensis*) as the type species. This genus belongs to the family Wickerhamomycetaceae of the order Saccharomycetales [1]. *Wickerhamomyces* species can reproduce both asexually and sexually. Through asexual reproduction, the species reproduce by budding and some species produce pseudohyphae and/or true hyphae. Alternatively, in sexual reproduction they produce hat-shaped or spherical ascospores with an equatorial ledge for sexual reproduction [1,2]. Most of the known *Wickerhamomyces* species can utilize various carbon sources, but not methanol or hexadecane. Nitrate utilization was observed in some species, while the diazonium blue B reaction was negative for all species. The predominant ubiquinone in the *Wickerhamomyces* species is CoQ-7 [1,3].

**Figure 1:**
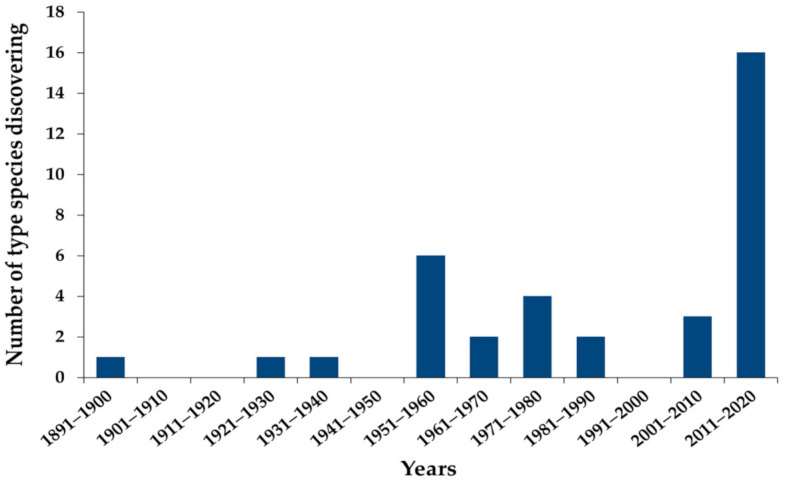
The discovering of *Wickerhamomyces* type species since 1891 till the present time.

**Figure 2:**
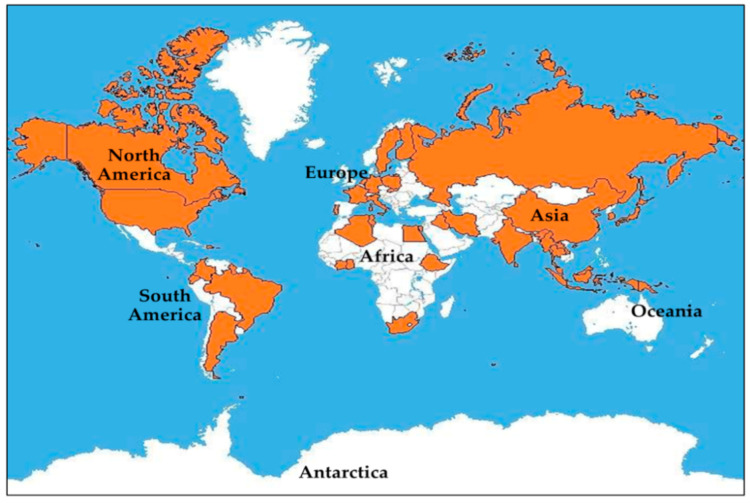
Global distribution of *Wickerhamomyces* species. The countries where *Wickerhamomyces* species have been discovered are indicated in orange color.

Most species of the genus *Wickerhamomyces* have been transferred from the genera *Candida*, *Hansenula*, *Pichia* and *Williopsis* based on phylogenetic analyses [1,2,4,5,6]. Currently, a total of 35 species have been accepted and recorded in the Index Fungorum [7]. A phylogenetic study of Arastehfar et al. [8] has suggested that *Pichai myanmarensis* [9] should be transferred to the genus *Wickerhamomyces*, but *W*. *myanmarensis* has remained invalidly published. Therefore, in this study, we have proposed the validation of this name for a new combination species. In this case, 35 type species of *Wickerhamomyces* and *P*. *myanmarensis* have been reported since 1891. It has been revealed that the highest number of the *Wickerhamomyces* species were discovered during the period of 2011–2020, followed by the period of 1951–1960 (6 species), the period of 1971–1980 (4 species) and the period of 2001–2010 (3 species) (Figure 1). An increasing trend with regard to the discovery of new species of *Wickerhamomyces* is expected to continue in the future. *Wickerhamomyces* species are widely distributed in tropical, subtropical, temperate and subpolar areas throughout the world (Figure 2). It has been reported that the highest number of *Wickerhamomyces* species were found in Asia (18 species), followed by Europe (12 species), South America (8 species), Africa (7 species), North America (3 species), Oceania (1 species) and Antartica (1 species) [7,8,9,10,11,12,13,14,15,16,17,18,19,20,21,22,23,24,25,26,27,28,29,30,31,32,33,34,35,36,37,38,39,40,41,42,43,44,45,46,47,48,49,50,51,52,53,54,55,56,57,58,59,60,61,62,63,64,65,66,67,68,69,70,71,72,73,74,75,76,77,78,79,80,81,82] (Table 1). Both *W*. *anomalus* and *W*. *onychis* are known to be from Asia, Africa, Europe and South America [13−35,54−59]. Moreover, *W*. *anomalus* and *W*. *rabaulenis* have been discovered in Antarctica (King George Island) [17] and Oceania (Papua New Guinea) [67], respectively. Recently, *W*. *psychrolipolyticus* has been discovered from Japan [65]. Consequently, *Wickerhamomyces* species have been successfully isolated from various habitats, as has been summarized in Table 1.

Many species of *Wickerhamomyces* have been used in a variety of industries including the medicinal, agricultural, biofuel, food and beverage industries, and a number of others [64]. Most previous studies have focused on different strains of *W*. *anomalus* for biotechnological applications. For example, the *W*. *anomalus* strains CBS261, HN006 and HN010 are capable of excessively producing ethyl acetate. As a result, this species has been used in the brewing of Baijiu (Chinese liquor) and in winemaking to improve the aroma and quality of the finished product [83,84,85].

*Wickerhamomyces anomalus* strains BS91 and DMKU-RP04 could effectively inhibit plant pathogenic fungi and have been used as a biological control agent in agriculture [86,87,88]. Notably, *W*. *anomalus* strains SDBR-CMU-S1-06 and Wa-32 have exhibited plant growth promotion potential by solubilizing insoluble minerals, producing indole-3-acitic acid (IAA) and siderophores, and by secreting various extracellular enzymes [16,89]. Moreover, most strains of *W*. *anomalus* are known to produce killer toxins that possess antimicrobial and larvicidal activities [90,91]. *Wickerhamomyces bovis* and *W*. *silvicola* have been observed to produce mycocin, which exhibited fungicidal activity [92,93]. In addition, *W*. *lynferdii* and *W*. *sydowiorum* have been recognized as relevant yeast species for the improvement of the fermentation processes for coffee cherries and cocoa, respectively [79,94]. Furthermore, *W*. *subpelliculosus* has been used as an alternative to baker’s yeast [95], while *W. chambardii* could produce amylase and cellulase enzymes that could be used to produce bioethanol from corn straw [96,97]. Previous studies have found that the biosurfactants produced by *W*. *anomalus* and *W*. *edaphicus* [47,98,99], the saturated fatty acids produced by *W. siamensis* [100], xylitol produced by *W*. *rabaulensis* [101], cellulase enzymes produced by *W*. *psychrolipolyticus* [65] and the extracellular polysaccharide produced by *W*. *mucosus* [102] could be applied in the bioremediation, biotechnological and cosmetic industries. Furthermore, these substances could also be employed in the production of detergents, food and various pharmaceuticals, as well as in the process of oil recovery enhancement.

On the other hand, some *Wickerhamomyces* species (e.g., *W*. *anomalus* and *W*. *lynferdii*) have been responsible for the spoilage of beer and bakery products [103,104,105,106]. Some cases of human infection caused by *W*. *anomalus*, *W*. *myanmarensis* and *W*. *onychis* have also been reported, but only with patients with serious illness [8,107,108,109,110,111]. Based on this evidence, *W*. *anomalus* has been labeled a biosafety level 1 organism by the European Food Safety Authority [112] and is considered safe for consumption by healthy individuals.

Currently, only eight *Wickerhamomyces* species, namely *W*. *anomalus*, *W*. *ciferrii*, *W*. *edaphicus*, *W*. *rabaulensis*, *W*. *siamensis*, *W*. *sydowiorum*, *W*. *tratensis* and *W*. *xylosicus*, have been reported in Thailand [4,8,16,17,19,20,46,70,78]. Accordingly, Thailand has been identified as a hotspot for unexpected novel species and the newly recorded discovery of many microorganisms [113,114]. In our previous investigation on yeasts in soil samples collected from Assam tea (*Camellia sinensis* var. *assamica*) plantations in northern Thailand [16], we obtained five yeast strains belonged to the genus *Wickerhamomyces* that represent potentially new species. In our present study, we have described them into two novel species. These two novel species are introduced based on their phenotypic (morphological, biochemical and physiological data) and molecular characteristics. To confirm their taxonomic status, phylogenetic relationship was determined by analysis of the combined sequence dataset of the D1/D2 domains of LSU and ITS sequences.

## 2. Materials and Methods

### 2.1. Yeast Strain

Five yeasts strains (SDBR-CMU-S2-02, SDBR-CMU-S2-06, SDBR-CMU-S2-14, SDBR-CMU-S2-17 and CMU-S3-15) isolated from soils of Assam tea (*C*. *sinensis* var. *assamica*) plantations in Thep Sadej, Doi Saket District, Chiang Mai Province and Sri Na Pan, Muang District, Nan Province, northern Thailand [16] were selected for this present study. All strains were deposited in the culture collection of the Sustainable Development of Biological Resources, Faculty of Science, Chiang Mai University (SDBR-CMU), Chiang Mai Province and Thailand Bioresource Research Center (TBRC), Pathum Thani Province, Thailand.

### 2.2. Yeast Identification

#### 2.2.1. Morphological Study

The morphological characteristics of yeast strains were determined according to established methods by Kurtzman et al. [2], Yarrow [3] and Limtong et al. [10]. Colony characters were observed on yeast extract-malt extract agar (YMA) after two days of incubation in darkness at 30 °C. Ascospore formation was investigated on YMA, 5% malt extract agar (MEA), potato dextrose agar (PDA) and V8 agar after incubation at 25 °C in the dark for four weeks. Micromorphological characteristics were examined under a light microscope (Nikon Eclipse Ni U, Tokyo, Japan). Size data of the anatomical structure (e.g., cells, pseudohyphae, asci and ascospores) were based on at least 50 measurements of each structure using the Tarosoft (R) Image Frame Work program.

#### 2.2.2. Biochemical and Physiological Studies

Biochemical and physiological characterizations of yeast strains was followed the previous studies [2,3,115]. Fermentation of carbohydrates including glucose, galactose, maltose, sucrose, trehalose, melibiose, lactose, raffinose, and xylose were performed. Additionally, assimilation tests for carbon (D-glucose, D-galactose, L-sorbose, N-acetyl glucosamine, D-ribose, D-xylose, L-arabinose, D-arabinose, rhamnose, sucrose, maltose, α,α-trehalose, α-methyl-D-glucoside, cellobiose, salicin, melibiose, lactose, raffinose, melezitose, inulin, soluble starch, glycerol, erythritol, ribitol, D-glucitol, D-mannitol, galactitol, myo-inositol, D-glucono-1,5-lactone, 2-ketogluconic acid, 5-ketogluconic acid, D-gluconate, D-glucuronate, D-galacturonic acid, DL-lactate, succinate, citrate, methanol, ethanol, and xylitol) and nitrogen compounds (ammonium sulfate, potassium nitrate, sodium nitrite, ethylamine hydrochloride, L-lysine, cadaverine, and creatine) were determined. Moreover, the effects of temperature on growth were examined by cultivation on YMA at temperature ranging from 15–45 °C and diazonium blue B reactions were tested [116].

#### 2.2.3. Molecular Study

Each yeast strain was grown in 5 mL of yeast extract-malt extract broth in 18 × 180 mm test tubes with shaking at 150 rpm on an orbital shaker in the dark for two days. Yeast cells were harvested by centrifugation at 11,000 rpm and washed three times with sterile distilled water. Genomic DNA was extracted from yeast cells using DNA Extraction Mini Kit (FAVORGEN, Taiwan) following the manufacturer’s protocol. The ITS region and D1/D2 domains of LSU gene were amplified by polymerase chain reactions (PCR) using ITS1/ITS4 primers [117] and NL1/NL4 primers [118], respectively. The amplification of both D1/D2 domains and ITS region process consisted of an initial denaturation at 95 °C for 5 min, followed by 35 cycles of denaturation at 95 °C for 30 s, annealing at 52 °C for 45 s, an extension at 72 °C for 1 min and 72 °C for 10 min on a peqSTAR thermal cycler (PEQLAB Ltd., UK). PCR products were checked and purified by a PCR clean up Gel Extraction NucleoSpin^®^ Gel and PCR Clean-up Kit (Macherey-Nagel, Germany). Final PCR products were sent to 1st Base Company Co., Ltd., (Kembangan, Malaysia) for sequencing. The obtained sequences were used to query GenBank via BLAST (http://blast.ddbj.nig.ac.jp/top-e.html, accessed on 25 August 2021).

Phylogenetic analysis was carried out based on the combined dataset of ITS and D1/D2 domains of LSU sequences. Sequences from this study along with those obtained from previous studies and the GenBank database were selected and provided in Table 2. Multiple sequence alignment was performed using MUSCLE [119]. A combination of D1/D2 domains of LSU and ITS alignment was deposited in TreeBASE under the study ID number 28785. A phylogenetic tree was constructed under maximum likelihood (ML) and Bayesian inference (BI) methods. The ML analysis was carried out using RAxML-HPC2 on XSEDE (8.2.10) in CIPRES Science Gateway V. 3.3 [120] using GTRCAT model with 25 categories and 1000 bootstrap (BS) replications. The optimum nucleotide substitution model was obtained using jModeltest v.2.3 [121] under the Akaike information criterion (AIC) method. The BI analysis was performed using MrBayes 3.2.6 software for Windows [122]. The selected optimal model of each gene is similar as GTR + I + G model. Six simultaneous Markov chains were run with one million generations and starting from random trees and keeping one tree every 100th generation until the average standard deviation of split frequencies was below 0.01. The value of burn-in was set to discard 25% of trees when calculating the posterior probabilities. Bayesian posterior probabilities (PP) were obtained from the 50% majority rule consensus of the trees kept. The tree topologies were visualized in FigTree v1.4.0 [123].

## 3. Results

### 3.1. Phylogenetic Results

**Figure 3:**
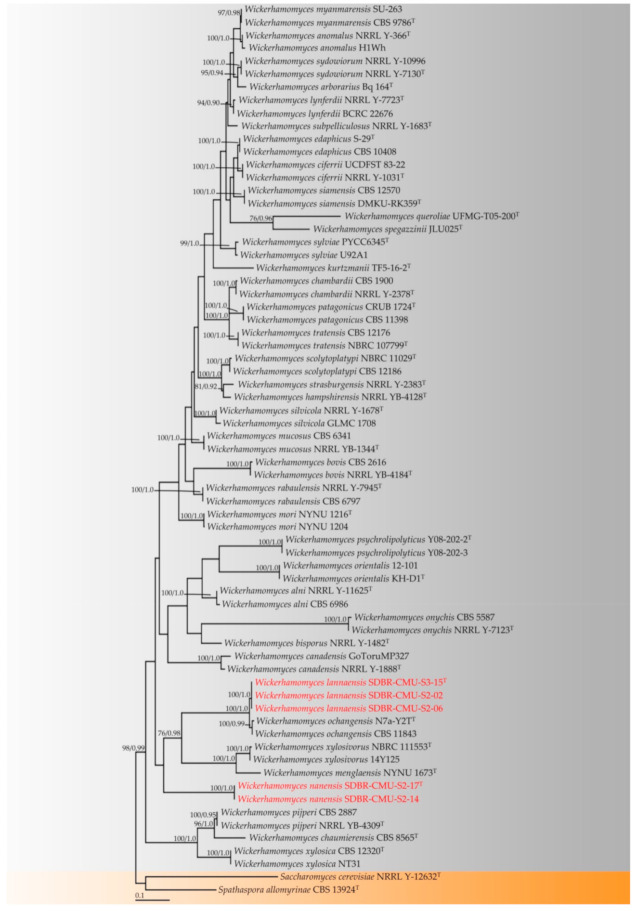
Phylogram derived from maximum likelihood analysis of 69 sequences of the combined ITS and D1/D2 sequences. *Saccharomyces cerevisiae* NRRL 12632 and *Spathaspora allomyrinae* CBS 13924 were used as the outgroup. The numbers above branches represent bootstrap percentages (**left**) and Bayesian posterior probabilities (**right**). Bootstrap values ≥ 75% and Bayesian posterior probabilities ≥ 0.90 are shown. Sequences obtained in this study are in red. Superscription “T” means the type strains.

The sequences of five yeast strains were deposited in the GenBank database (Table 2). The alignment of a combination of ITS and D1/D2 domains of the LSU genes contained 1544 characters including gaps (ITS: 1−823 and D1/D2 domains of LSU: 824−1544). RAxML analysis of the combined dataset yielded a best scoring tree with a final ML optimization likelihood value of −12,120.4323. The matrix contained 776 distinct alignment patterns with 42.33% undetermined characters or gaps. Estimated base frequencies were recorded as follows: A = 0.2730, C = 0.1821, G = 0.2603, T = 0.2844; substitution rates AC = 1.0574, AG = 2.0209, AT = 1.4684, CG = 0.6712, CT = 4.4165, GT = 1.0000. The gamma distribution shape parameter alpha was equal to 0.2698 and the Tree-Length was equal to 4.4075. In addition, the final average standard deviation of the split frequencies at the end of the total MCMC generations was calculated as 0.00638 through BI analysis. Phylograms of the ML and BI analyses were similar in terms of topology (data not shown). Therefore, the phylogram obtained from the ML analysis was selected and presented for this study. The phylogram was comprised of 67 sequences of *Wickerhamomyces* strains (including 37 type strains obtained from either previous studies or the present study) and two sequences (*Saccharomyces cerevisiae* NRRL 12632 and *Spathaspora allomyrinae* CBS 13924) of the outgroup (Figure 3). Our phylogenetic analysis separated *Wickerhamomyces* by different species based on different topologies. Our analysis confirmed that *W. myanmarensis* (previously known as *P. myanmarensis*) belonged to the genus *Wickerhamomyces* according to the phylogenetic results of Arastehfar et al. [8] and Shimizu et al. [65]. Moreover, a phylogram clearly separated our yeast strains into two monophyletic clades with high support values (BS = 100% and PP = 1.0). The results indicated that our two yeast strains, SDBR-CMU-S2-17 and SDBR-CMU-S2-14 (introduced as *W. nanensis*), were clearly distinguished from the previously known species of *Wickerhamomyces*. Moreover, three yeast strains in this study, SDBR-CMU-S2-02, SDBR-CMU-S2-15, and CMU-S3-06 (described here as *W. lannaensis*) formed a sister clade to *W. ochangensis* with high support (BS = 100% and PP = 1.0).

### 3.2. Taxonomic Description of New Species

#### 3.2.1. *Wickerhamomyces lannaensis* S. Nundaeng, J. Kumla, N. Suwannarach and S. Lumyong, sp. nov. (Figure 4)

Mycobank No.: 841356

Etymology: “*lannaensis*” refers to Lanna kingdom the historic name of northern Thailand, the collection locality of the type strain of the species.

Holotype: Thailand, Chiang Mai Province, Thep Sadej, Doi Saket District, in soil from Assam tea (*C*. *sinensis* var. *assamica*) plantation, May 2017, J. Kumla and N. Suwannarach, (holotype SDBR-CMU-S3-15^T^, culture ex-type TBRC 15533)

Description: The streak culture on YMA after two days at 30 °C is circular from (1–2 mm in diameter), white to cream color, smooth surface, dull-shining, entire margin, and raised elevation. After growth on YMA at 30 °C for two days, the cells are spheroidal to short ovoidal (3.6–3.8 × 2.4–2.6 µm), occur singly or in budding pairs. Pseudohyphae and true hyphae were absent. Ascospores were not obtained for individual strains and strain pairs on YMA, 5% MEA, PDA and V8 agar after incubation at 30 °C for one month. Urea hydrolysis and diazonium blue B reactions are negative. Fermentation tests, glucose is delayed positive, but galactose, maltose, sucrose, trehalose, melibiose, lactose, raffinose, and xylose are negative. D-glucose, D-xylose, rhamnose, cellobiose, salicin, inulin (weak), glycerol, D-glucitol, D-mannitol, D-glucono-1,5-lactone, D-gluconate, DL-lactate (weak), succinate, and ethanol are assimilated. No growth was observed in L-sorbose, N-acetyl glucosamine, D-ribose, L-arabinose, D-arabinose, sucrose, maltose, α,α-trehalose, α-methyl-D-glucoside, melibiose, lactose, raffinose, melezitose, soluble starch, erythritol, ribitol, galactitol, myo-inositol, 2-ketogluconic acid, 5-ketogluconic acid, D-glucuronate, D-galacturonic acid, citrate, methanol, and xylitol. For the assimilation of nitrogen compounds, growth on ammonium sulfate, potassium nitrate, sodium nitrite, ethylamine HCl, cadaverine, and creatine (weak) are positive and on L-lysine is latent positive.

Growth in the vitamin-free medium is weak positive. Growth was observed at 15 °C and 30 °C, but not at 35, 37, 40, 42 and 45 °C. Growth in the presence of 50% glucose is positive, but growth in the presence of 0.01% cycloheximide, 0.1% cycloheximide, 60% glucose, 10% NaCl with 5% glucose and 15% NaCl with 5% glucose are negative. Acid formation is negative.

Additional strains examined: Thailand, Nan Province, Muang District, Sri Na Pan, in soil from Assam tea (*C*. *sinensis* var. *assamica*) plantation, September 2016, J. Kumla and N. Suwannarach, SDBR-CMU-S2-02, SDBR-CMU-S2-06.

GenBank accession numbers: holotype SDBR-CMU-S3-15 (D1/D2: MT639220, ITS: OK135750); additional strains SDBR-CMU-S2-02 (D1/D2: MT623569, ITS: OK135752) and SDBR-CMU-S2-06 (D1/D2: MT613722, ITS: OK135753).

Note: Based on phylogenetic analyses, *W. lannaensis* formed a monophyletic clade in a well-supported clade and was found to be closely related to *W. ochangensis* (Figure 3). *Wickerhamomyces lannaensis* can be distinguished from *W. ochangensis* by its ability to assimilate inulin and creatine and its growth in 50% glucose medium [11]. Additionally, *W. ochangensis* was able to grow at a temperature of 37 °C, while *W. lannaensis* could not grow at 37 °C [11].

#### 3.2.2. *Wickerhamomyces nanensis* J. Kumla, S. Nundaeng, N. Suwannarach and S. Lumyong, sp. nov. (Figure 5)

Mycobank No.: 841357

Etymology: “*nanensis*” refers to Nan Province of Thailand, the collection locality of the type strain of the species.

Holotype: Thailand, Nan Province, Muang District, Sri Na Pan, in soil from Assam tea (*C. sinensis* var. *assamica*) plantation, September 2016, J. Kumla, N. Suwannarach and S. Khuna, (holotype SDBR-CMU-S2-17^T^, culture ex-type TBRC 15534)

Description: The streak culture on YMA after two days at 30 °C is circular from (1–2 mm in diameter), white to cream color, smooth surface, dull-shining, entire margin, and raised elevation. After growth on YMA at 30 °C for two days, the cells are spheroidal to short ovoidal (3.8–4.0 × 2.4–2.5 µm), occur singly or in budding pairs. Pseudohyphae (4.8–6.9 × 2.2–2.9 µm) were produced in Dalmau plate culture on 5% MEA and PDA after 7 days at 25 °C, but true hyphae are not obtained. Ascospores were not observed for individual strains and strain pairs on YMA, 5% MEA, PDA and V8 agar after incubation at 30 °C for one month. Urea hydrolysis and diazonium blue B reactions are negative. Fermentation test, glucose is delayed positive, but galactose, maltose, sucrose, trehalose, melibiose, lactose, raffinose, and xylose are not positive. D-glucose, D-galactose, cellobiose, salicin, glycerol, D-mannitol, D-glucono-1,5-lactone, DL-lactate (weak), succinate, citrate, and ethanol are assimilated. No growth was observed in L-sorbose, N-acetyl glucosamine, D-ribose, D-xylose, L-arabinose, D-arabinose, rhamnose, sucrose, maltose, α,α-trehalose, α-methyl-D-glucoside, melibiose, lactose, raffinose, melezitose, inulin, soluble starch, erythritol, ribitol, D-glucitol, galactitol, myo-inositol, 2-ketogluconic acid, 5-ketogluconic acid, D-gluconate, D-glucuronate, D-galacturonic acid, methanol, and xylitol. For the assimilation of nitrogen compounds, growth on ammonium sulfate, potassium nitrate (weak), sodium nitrite (weak), ethylamine HCl, l-lysine, and creatine (slow) are positive, but cadaverine is not. Growth in the vitamin-free medium is weak. Growth was observed at 15 °C and 30 °C, but not at 35, 37, 40, 42 and 45 °C. Growth in the presence of 50% glucose and acid formation are positive, but growth in the presence of 0.01% cycloheximide, 0.1% cycloheximide, 60% glucose, 10% NaCl with 5% glucose, and 15% NaCl with 5% glucose are negative.

Additional strain examined: Thailand, Nan Province, Muang District, Sri Na Pan, in soil from Assam tea (*C. sinensis* var. *assamica*) plantation, September 2016, J. Kumla and N. Suwannarach, SDBR-CMU-S2-14.

GenBank accession numbers: holotype SDBR-CMU-S2-17 (D1/D2: MT613875, ITS: OK143510); additional strain SDBR-CMU-S2-14 (D1/D2: MT623569, ITS: OK143511).

Note: Several morphological and biochemical characteristics of *W. nanensis* were similar to *W. chambardii*. However, *W. chambardii* differed from *W. nanensis* by its ascospore formation and could not assimilate D-mannitol [2]. Phylogenetic analyses clearly separated *W. nanensis* and *W. chambardii* as different species. Moreover, *W. nanensis* formed a monophyletic clade in a well-supported clade and was separated from other *Wickerhamomyces* species (Figure 3).

### 3.3. New Combination

*Wickerhamomyces myanmarensis* (Nagats., H. Kawas. and T. Seki) J. Kumla, N. Suwannarach and S. Lumyong, comb. nov.

Mycobank No.: 841356

Basionym: *Pichia myanmarensis* Nagats., H. Kawas. and T. Seki, Int. J. Syst. Evol. Microbiol. 55: 1381, 2005.

Note: The combined ITS and D1/D2 phylogenetic analyses indicated that the type species, *P. myanmarensis*, belongs to the genus *Wickerhamomyces* and has a close phylogenetic relationship with *W. anomalus* (Figure 3). Accordingly, the phylogenetic results of Arastehfar et al. [8] and Shimizu et al. [65] found that *P. myanmarensis* was placed within the genus *Wickerhamomyces*.

### 3.4. Key to Species of Wickerhamomyces

A key to the identification of the *Wickerhamomyces* species introduced in the present study was derived from the key described by Kurtzman et al. [2]. Key characteristics are shown in Table 3.

1. a. Melibiose is assimilated………………...……………………..…..…..…………………2
   b. Melibiose is not assimilated………..……………………………..…..….…….….….…7
2. (1) a. Raffinose is assimilated………….…….…..…….….….….….……..………………3
      b. Raffinose is not assimilated………………….…………………...….*W. kurtzmanii*
3. (2) a. Citrate is assimilated………………………….……….………………….…………4
      b. Citrate is not assimilated…………………….…….……………......….*W. orientalis*
4. (3) a. Ribitol is assimilated………………...………….……………………………………5
      b. Ribitol is not assimilated………………...………………………..…*W. spegazzinii*
5. (4) a. Growth at 37 °C……………………………………………….….…….*W. edaphicus*
      b. Growth is absent at 37 °C…………………………...…………..…….……………6
6. (5) a. Ascospores observed on 5% MEA……………………………....…*W. sydowiorum*
      b. Ascospores not observed on 5% MEA…………….………..…….…*W. arborarius*
7. (1) a. Raffinose is assimilated…………………………….….……………………………8
      b. Raffinose is not assimilated………………………………..………..…………….19
8. (7) a. Nitrate is assimilated………………….……………………….………...………….9
      b. Nitrate is not assimilated…………………………………………………….……15
9. (8) a. L-Rhamnose is assimilated……………………………………………….……….10
         b. L-Rhamnose is not assimilated………………………….………………………..12
10. (9) a. L-Arabinose is assimilated…………………………..………………...….*W. ciferrii*
          b. L-Arabinose is not assimilated…………………………..………………….……11
11. (10) a. Sucrose is assimilated……………………………...……….*W. psychrolipolyticus*
          b. Sucrose is not assimilated…………………………….……...…...*W. xylosivorus*
12. (9) a. Growth in vitamin-free medium…………………………………………………13
        b. Growth is absent in vitamin-free medium……….…..………..*W. subpelliculosus*
13. (12) a. Soluble starch is assimilated…………………...……………………………….14
       b. Soluble starch is not assimilated………………………….………....*W. lynferdii*
14. (13) a. D-Arabinose is assimilated……………..……..….……………*W. myanmarensis*
       b. D-Arabinose is not assimilated…………………..…………………*W. anomalus*
15. (8) a. Ribitol is assimilated…………………………………………………………….16
       b. Ribitol is not assimilated…………………………………….…………….……17
16. (15) a. Galactose is assimilated……………………….....…………….*W. strasburgensis*
       b. Galactose is not assimilated……………………..………..………*W. rabaulensis*
17. (15) a. Growth in vitamin-free medium……….………....…………..…*W. patagonicus*
       b. Growth is absent in vitamin-free medium…………………….……………...18
18. (17) a. Citrate is assimilated……………………….…………...….….………*W. onychis*
       b. Citrate is not assimilated………………………..…………..………*W. siamensis*
19. (7) a. 2-Keto-D-gluconate is assimilated……………………………………….……….20
     b. 2-Keto-D-gluconate is not assimilated……………………………...……………21
20. (19) a. D-Glucitol is assimilated…………………………………….……….*W. mucosus*
       b. D-Glucitol is not assimilated……………………….……………….*W. xylosicus*
21. (19) a. D-Arabinose is assimilated………………………………..………………………22
       b. D-Arabinose is not assimilated…………………………..…………………….23
22. (21) a. Growth at 37 °C……….………………...………………………...…….*W. sylviae*
       b. Growth is absent at 37 °C……………….…………….………………….*W. mori*
23. (21) a. Galactose is assimilated……………………………………..…………...……..24
       b. Galactose is not assimilated………………...…………………....…………….27
24. (23) a. L-Arabinose is assimilated……………….……………………..…….*W. silvicola*
       b. L-Arabinose is not assimilated………………...…………………....…...…….25
25. (24) a. Sucrose is assimilated…………….………………….………….*W. scolytoplatypi*
       b. Sucrose is not assimilated…………………….………………..……….………26
26. (24) a. D-Mannitol is assimilated……………………..………….…….……*W. nanensis*
       b. D-Mannitol is not assimilated………………..….……….…....….*W. chambardii*
27. (23) a. L-Sorbose is assimilated………………….……...…..…………..……...*W. pijperi*
       b. L- Sorbose is not assimilated……………………….………….….……………28
28. (27) a. D-Xylose is assimilated……………………………………………...………….29
       b. D-Xylose is not assimilated……………….…………..….………….*W. tratensis*
29. (28) a. Sucrose is assimilated……………………………...……………………………30
       b. Sucrose is not assimilated……………………………...……………….………36
30. (29) a. Cellobiose is assimilated……………………………………..………………....31
       b. Cellobiose is not assimilated…………..…………………………….*W. queroliae*
31. (30) a. D-Glucitol is assimilated…………………………………………….………….32
       b. D-Glucitol is not assimilated…………………….…………….*W. chaumierensis*
32. (31) a. Growth at 37 °C………………………………….……………………………....33
       b. Growth is absent at 37 °C………………………………...…………….………34
33. (32) a. L-Arabinose is assimilated……………………….....………..………….*W. bovis*
       b. L-Arabinose is not assimilated……………..…………….……….*W. canadensis*
34. (32) a. Nitrate is assimilated……………………………………………...…………….35
       b. Nitrate is not assimilated…………………………..……...…...*W. hampshirensis*
35. (34) a. True hyphae are formed……………..……………………………….*W. bisporus*
       b. True hyphae are not formed…………………………………...…………*W. alni*
36. (29) a. Citrate is assimilated……………………………...……..………..*W. menglaensis*
       b. Citrate is not assimilated……………………………….………………………37
37. (36) a. Growth at 37 °C……………………..………..……..…..…………*W. ochangensis*
       b. Growth is absent at 37 °C………………..………...…..…….…….*W. lannaensis*

## 4. Discussion

Traditional methods of identification and characterization for the *Wickerhamomyces* species are based primarily on phenotypical characteristics. These are further recognized as relevant morphological, biochemical, and physiological characteristics [41,128]. However, identification can be difficult because some species have similar appearances, and some biochemical characteristics are consistent across a number of species. In accordance with this evidence, previous species of *Wickerhamomyces* were originally classified into various yeast genera [1,2,4,5,50,70,75]. In 2008, the genus *Wickerhamomyces* was proposed by Kurtzman et al. [1], wherein this genus was clearly separated from other yeast genera based on phylogenetic evidence. Subsequently, some previously identified species were then transferred from the genera *Candida*, *Hansenula*, *Pichia,* and *Williopsis* [1,4,5,50,54,70]. Therefore, molecular phylogenetic analysis is necessary to concretely identify the *Wickerhamomyces* species. Species of the genus *Wickerhamomyces* are known to be widely distributed throughout the world and have been isolated from various habitats as shown in Table 1. Prior to conducting our study, *Wickerhamomyces* consisted of 35 accepted and published species according to molecular phylogenetic analysis. Our phylogenetic results were similar to those of Arastehfar et al. [8] and Shimizu et al. [65] who indicated that *P*. *myanmarensis* should be placed in the genus *Wickerhamomyces*. Consequently, we have proposed that this yeast species be named *W*. *myanmarensis*.

Yeast diversity has been investigated in various habitats throughout different regions of Thailand [5,10,15,16,18,19,46,70,78]. *Wickerhamomyces anomalus* was first species reported in Thailand in 2002 [20]. In 2009, the first new species, *W. edaphicus* has been discovered in Thailand [10]. Until now, a total of eight *Wickerhamomyces* species have been found [5,10,20,46,68,70,78,82]. However, *W*. *siamensis*, *W. tratensis,* and *W*. *xylosicus* were only known to be from Thailand [5,70,82]. In this study, two new *Wickerhamomyces* species, namely *W*. *lannaensis* and *W*. *nanensis*, that were isolated from soil collected from Assam tea plantations in northern Thailand were proposed based on identification through molecular phylogenetic and phenotypic (morphological, biochemical, and physiological characteristics) analyses. Therefore, effective identification of the *Wickerhamomyces* species has increased the number of species found in Thailand to 10 species and has led to 38 global species. This present discovery has increased the number of species of yeast known to be from Thailand and is considered important in terms of stimulating deeper investigations of yeast varieties in Thailand. Ultimately, these findings will help researchers gain a better understanding of the distribution and ecology of *Wickerhamomyces*.

Many species of the genus *Wickerhamomyces* have been investigated, and some strains have been used in a variety of biotechnology, food, and beverage industries, as well as in medical and agricultural fields [83,84,85,86,87,88,89,90,91,92,93,94,95,96,97,98,99,100,101,102]. Despite the fact that many *Wickerhamomyces* species can survive in a variety of environments, climate change has had an impact on both the terrestrial biome and the aquatic environment. These environments are known to serve as habitats for a number of microorganisms [130,131,132,133,134] and may have an impact on the global diversity and distribution of *Wickerhamomyces*. Therefore, in addition to studying the diversity and distribution of newly identified species, future research should focus on the effects of climate change on *Wickerhamomyces*.

## Figures and Tables

**Figure 4:**
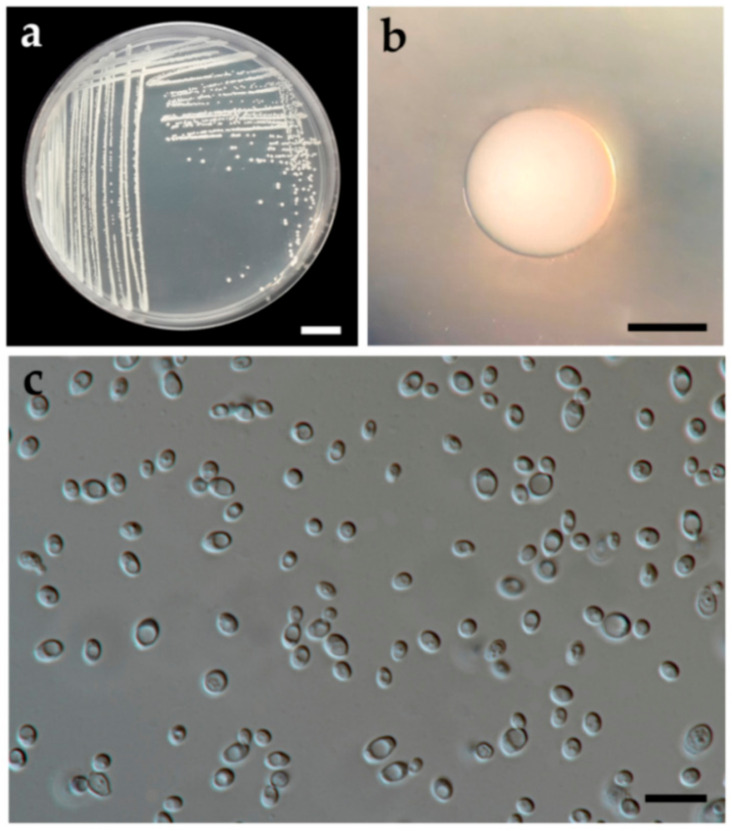
*Wickerhamomyces lannaensis* (holotype SDBR-CMU-S3-15). Culture (**a**), single colony (**b**) and budding cells (**c**) on YMA after two days at 30 °C. Scale bar a and b = 10 cm, c = 10 µm.

**Figure 5:**
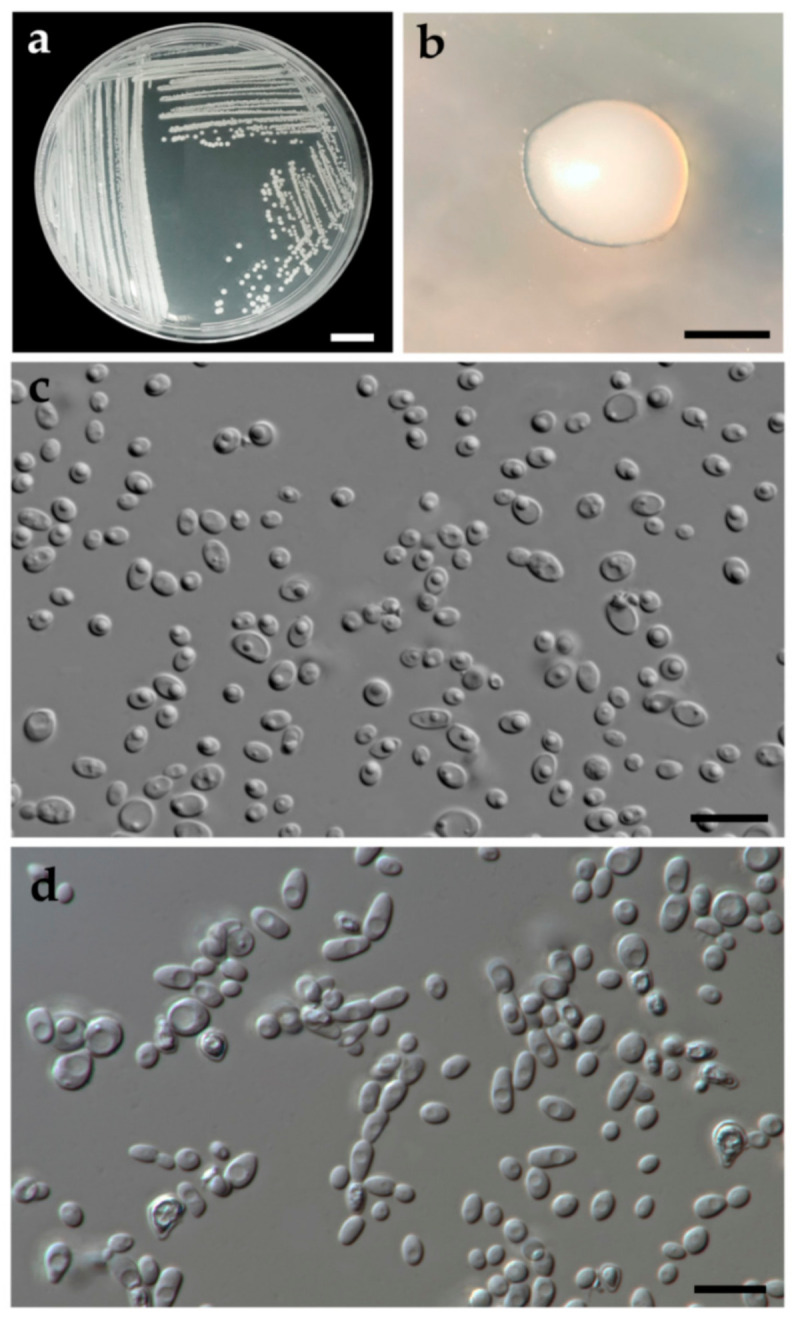
*Wickerhamomyces nanensis* (holotype SDBR-CMU-S3-15). Culture (**a**), single colony (**b**) and budding cells (**c**) on YMA after two days at 30 °C. Pseudohypahae (**d**) on 5% MAE agar after 7 days at 25 °C. Scale bar a and b = 10 cm, c and d = 10 µm.

**Table 1:** Global distribution and isolation sources of *Wickerhamomyces* species.

No.	Spices	Known Distribution	Isolation Source	Reference
1	*Wickerhamomyces alni* (Phaff, M.W. Mill. and M. Miranda) Kurtzman, Robnett and Bas.-Powers	Canada	Exudate of *Alnus rubra*	[12]
2	*Wickerhamomyces anomalus* (E.C. Hansen) Kurtzman, Robnett and Bas.-Powers	Algeria, Brazil, China, Colombia, Ethiopia, India, Iraq, King George Island, Lao, Russia, Slovakia Sweden and Thailand	Process of beer and wine, phylloplane, soil, water, coral reefs, Thai traditional alcoholic starter, mangrove forest, fermented food, flowers, fruits, fermented grains, coffee processing, wastewater treatment plant, Colombian fermented beans and Brazilian spirit	[13,14,15,16,17,18,19,20,21,22,23,24,25,26,27,28,29,30,31,32,33,34,35]
3	*Wickerhamomyces arborarius* S.A. James, E.J. Carvajal, Barahona, T.C. Harr., C.F. Lee, C.J. Bond and I.N. Roberts	Ecuador	Flower	[36]
4	*Wickerhamomyces bisporus* (O. Beck) Kurtzman, Robnett and Bas.-Powers	Finland, France and USA	*Platypus compositus*, phoretic mites on *Ips typographus* and bark beetles (*Dendroctonus*)	[37,38,39]
5	*Wickerhamomyces bovis* (Uden and Carmo Souza) Kurtzman, Robnett and Bas.-Powers	Portugal	Caecum of feral cattle (*Bos taurus*)	[40]
6	*Wickerhamomyces canadensis* (Wick.) Kurtzman, Robnett and Bas.-Powers	Canada	Beetle frass from *Pinus resinosa*	[41]
7	*Wickerhamomyces chambardii* (C. Ramírez and Boidin) Kurtzman, Robnett and Bas.-Powers	France	Chestnut	[42]
8	*Wickerhamomyces chaumierensis* M. Groenew., V. Robert and M.T. Sm.	Guyana	Surface of flower	[43]
9	*Wickerhamomyces ciferrii* (Lodder) Kurtzman, Robnett and Bas.-Powers	Dominican Republic, USA and Thailand	Fruit of *Dipteryx odorata* and male olive fruit fly (*Bactrocera oleae*)	[44,45,46]
10	*Wickerhamomyces edaphicus* Limtong, Yongman., H. Kawas. and Fujiyama	India and Thailand	Forest and mangrove soils	[4,47]
11	*Wickerhamomyces hampshirensis* (Kurtzman) Kurtzman, Robnett and Bas.-Powers	USA	Frass of cut and dead of *Quercus* and beetle (*Xyloterinus politus*)	[48,49]
12	*Wickerhamomyces kurtzmanii* A.H. Li, Y.G. Zhou and Q.M. Wang	China	Crater lake water	[6]
13	*Wickerhamomyces lynferdii* (Van der Walt and Johannsen) Kurtzman, Robnett and Bas.-Powers	South Africa	Soil	[50]
14	*Wickerhamomyces menglaensis* F.L. Hui and L.N. Huang	China	Rotting wood	[51]
15	*Wickerhamomyces mori* F.L. Hui, Liang Chen, X.Y. Chu, Niu and T. Ke	China	Gut of larvae of wood-boring insect on trunk of *Morus alba*	[52]
16	*Wickerhamomyces mucosus* (Wick. and Kurtzman) Kurtzman, Robnett and Bas.-Powers	USA	Soil	[53]
17	*Wickerhamomyces myanmarensis* (Nagats., H. Kawas. and T. Seki) J. Kumla, N. Suwannarach and S. Lumyong	Iran and Myanmar	Palm sugar in rum distiller, and blood and central venous catheter of patients	[8,9]
18	*Wickerhamomyces ochangensis* K.S. Shin	South Korea	Soil of potato field	[11]
19	*Wickerhamomyces onychis* (Yarrow) Kurtzman, Robnett and Bas.-Powers	Brazil, Ethiopia, Iraq, Malaysia, Netherlands, Poland and Tunisia	Nail infection of *Homo sapiens*, fermented food, cocoa beans, grape and tomato during spontaneous fermentation, and soil	[23,54,55,56,57,58,59]
20	*Wickerhamomyces orientalis* Sipiczki, S. Nasr, H.D.T. Nguyen and Soudi	Iran and Sri Lanka	Fruits and rhizosphere soil	[27,60]
21	*Wickerhamomyces patagonicus* V. de García, Brizzio, C.A. Rosa, Libkind and Van Broock	Argentina	Sap exudate on cut branches of *Nothofagus dombeyi* and glacier meltwater river	[61]
22	*Wickerhamomyces pijperi* (Van der Walt & Tscheuschner) Kurtzman, Robnett and Bas.-Powers	Egypt, Ghana and South Africa	Buttermilk, cocoa fermentation and orange juice	[62,63,64]
23	*Wickerhamomyces psychrolipolyticus* Y. Shimizu and K. Konno	Japan	Soil	[65]
24	*Wickerhamomyces queroliae* C.A. Rosa, P.B. Morais, Lachance and Pimenta	Brazil	Larva of *Anastrepha mucronata* from fruit of *Peritassa campestris*	[66]
25	*Wickerhamomyces rabaulensis* (Soneda and S. Uchida) Kurtzman, Robnett and Bas.-Powers	Ethiopia, Papua New Guinea and Thailand	Excreta of snail, soils, decaying agricultural residues, decaying leaves and tree bark, and fermented food	[23,67,68]
26	*Wickerhamomyces scolytoplatypi* Ninomiya	Japan	Gallery of beetles (*Scolytoplatypus shogun*) in *Fagus crenata*	[69]
27	*Wickerhamomyces siamensis* Kaewwich., Yongman., H. Kawas. and Limtong	Thailand	Phylloplane of *Saccharum officinarum*	[70]
28	*Wickerhamomyces silvicola* (Wick.) Kurtzman, Robnett and Bas.-Powers	Germany, South Korea and USA	Flowers, gum of *Prunus serotina* and *Prunus* wood	[41,71,72]
29	*Wickerhamomyces spegazzinii* Masiulionis and Pagnocca	Argentina	The fungus garden of an attine ant nest (*Acromyrmex lundii*)	[73]
30	*Wickerhamomyces strasburgensis* (C. Ramírez and Boidin) Kurtzman, Robnett and Bas.-Powers	France	On leather tanned by vegetable means	[74]
31	*Wickerhamomyces subpelliculosus* (Kurtzman) Kurtzman, Robnett and Bas.-Powers	Egypt and USA	Fermenting cucumber brines, gut of honey bee and molasses	[75,76]
32	*Wickerhamomyces sydowiorum* (D.B. Scott and Van der Walt) Kurtzman, Robnett and Bas.-Powers	Brazil, Ivory Coast, South Africa and Thailand	Frass of *Sinoxylon ruficorne* in dead *Combretum apiculatum*, decayed plant leaf, fermented cocoa, honey, sand and water	[59,77,78,79,80]
33	*Wickerhamomyces sylviae* Moschetti and J.P. Samp.	Italy	Cloaca of migratory birds (*Sylvia communis*)	[81]
34	*Wickerhamomyces tratensis* Nakase, Jindam., Am-In, Ninomiya and H. Kawas.	Thailand	Flower of mangrove apple (*Sonneratia caseolaris*)	[82]
35	*Wickerhamomyces xylosicus* Limtong, Nitiyon, Kaewwich., Jindam., Am-In and Yongman	Thailand	Soil	[5]
36	*Wickerhamomyces xylosivorus* R. Kobay., A. Kanti and H. Kawas.	Indonesia	Decayed wood	[4]

**Table 2:** DNA sequences used in the molecular phylogenetic analysis. Type strains indicated by “^T^”.

Yeast Species	Strain	GenBank Acession Number		Reference
		ITS	D1/D2	
*Wickerhamomyces alni*	NRRL Y-11625^T^	‒	EF550294	[1]
	CBS 6986	NR154966	KY110065	[124]
*Wickerhamomyces anomalus*	NRRL Y-366^T^	‒	EF550341	[1]
	H1Wh	JQ857021	JQ856997	[17]
*Wickerhamomyces arborarius*	Bq 164^T^	NR_55000	FN908198	[36]
*Wickerhamomyces bisporus*	NRRL Y-1482^T^	‒	EF550296	[1]
*Wickerhamomyces bovis*	NRRL YB-4184^T^	‒	EF550298	[1]
	CBS 2616	NR154968	KY110109	[124]
*Wickerhamomyces canadensis*	NRRL Y-1888^T^	‒	EF550300	[1]
	GoToruMP327	EF093299	EF016107	[125]
*Wickerhamomyces chambardii*	NRRL Y-2378^T^	‒	EF550344	[1]
	CBS 1900	NR154969	KY110114	[124]
*Wickerhamomyces chaumierensis*	CBS 8565^T^	HM156503	HM156533	[43]
*Wickerhamomyces ciferrii*	NRRL Y-1031^T^	‒	EF550339	[1]
	UCDFST 83-22	MH153583	MH130275	[126]
*Wickerhamomyces edaphicus*	S-29^T^	AB436771	AB436763	[10]
	CBS 10408	KY105904	KY110120	[124]
*Wickerhamomyces hampshirensis*	NRRL YB-4128^T^	‒	EF550334	[1]
*Wickerhamomyces kurtzmanii*	TF5-16-2^T^	MK573939	MK573939	[6]
***Wickerhamomyces lannaensis***	**SDBR-CMU-S3-15^T^**	**OK135750**	**MT639220**	**This study,** [16]
	**SDBR-CMU-S2-02**	**OK135752**	**MT623569**	**This study**, [16]
	**SDBR-CMU-S2-06**	**OK135753**	**MT613722**	**This study**, [16]
*Wickerhamomyces lynferdii*	NRRL Y-7723^T^	EF550342	EF550342	[1]
	BCRC 22676	NR111798	‒	[127]
*Wickerhamomyces menglaensis*	NYNU 1673^T^	KY213818	KY213812	[51]
*Wickerhamomyces mori*	NYNU 1216^T^	JX204288	JX204287	[52]
	NYNU 1204	JX292100	JX292099	[52]
*Wickerhamomyces mucosus*	NRRL YB-1344^T^	‒	EF550337	[1]
	CBS 6341	Z93877	KY110124	[124]
*Wickerhamomyces myanmarensis*	CBS 9786^T^	‒	AB126678	[9]
	SU-263	MH236221	MH236219	[8]
***Wickerhamomyces nanensis***	**SDBR-CMU-S2-17^T^**	**OK143510**	**MT613875**	**This study**, [16]
	**SDBR-CMU-S2-14**	**OK143511**	**MT623571**	**This study**, [16]
*Wickerhamomyces ochangensis*	N7a-Y2^T^	NR154971	HM485464	[11]
	CBS 11843	KY105909	‒	[124]
*Wickerhamomyces onychis*	NRRL Y-7123^T^	‒	EF550279	[1]
	CBS 5587	KY105910	KY110125	[124]
*Wickerhamomyces orientalis*	KH-D1^T^	KF938677	KF938676	[60]
	12-101	KU253704	KU253703	[60]
*Wickerhamomyces patagonicus*	CRUB 1724^T^	FJ793131	FJ666399	[61]
	CBS 11398	NG057185	KY110126	[124]
*Wickerhamomyces pijperi*	NRRL YB-4309^T^	‒	EF550335	[1]
	CBS 2887	HM156502	KY110127	[124]
*Wickerhamomyces psychrolipolyticus*	Y08-202-2^T^	‒	LC333101	[65]
	Y08-202-2	‒	LC333102	[65]
*Wickerhamomyces queroliae*	UFMG-T05-200^T^	EU580140	EU580140	[66]
*Wickerhamomyces rabaulensis*	NRRL Y-7945^T^	‒	EF550303	[1]
	CBS 6797	KY105914	KY110128	[124]
*Wickerhamomyces scolytoplatypi*	NBRC 11029^T^	‒	AB534166	[69]
	CBS 12186	KY105915	KY110130	[124]
*Wickerhamomyces siamensis*	DMKU-RK359^T^	NR111029	AB714248	[70]
	CBS 12570	KY105916	KY110131	[124]
*Wickerhamomyces silvicola*	NRRL Y-1678^T^	‒	EF550302	[1]
	GLMC 1708	MT156140	MT156324	[71]
*Wickerhamomyces spegazzinii*	JLU025^T^	KJ832072	KJ832071	[73]
*Wickerhamomyces strasburgensis*	NRRL Y-2383^T^	‒	EF550333	[1]
*Wickerhamomyces subpelliculosus*	NRRL Y-1683^T^	NR111336	EF550340	[1,127]
*Wickerhamomyces sydowiorum*	NRRL Y-7130^T^	NR138219	EF550343	[1,36]
	NRRL Y-10996	FR690145	FR690073	[36]
*Wickerhamomyces sylviae*	PYCC6345^T^	‒	KF240728	[81]
	U92A1	‒	KF240729	[81]
*Wickerhamomyces tratensis*	NBRC 107799^T^	AB607029	AB607028	[82]
	CBS 12176	KY105935	KY110150	[124]
*Wickerhamomyces xylosica*	CBS 12320^T^	NR160310	AB557867	[5]
	NT31	AB704715	NG064304	[5]
*Wickerhamomyces xylosivorus*	NBRC 111553^T^	NR155013	LC202858	[4]
	14Y125	‒	NG057186	[4]
*Saccharomyces cerevisiae*	NRRL Y-12632^T^	AY046146	JQ689017	[128]
*Spathaspora allomyrinae*	CBS 13924^T^	KP054268	KP054267	[129]

Note: sepecies obtained in this study are in bold.

**Table 3:** Key characteristics of species assigned to the genus *Wickerhamomyces*.

Species	Growth in/at *																				Ascospores on 5% MEA	True Hyphae
	Ga	Sor	_D_Xy	_L_Ar	_D_Ar	Rh	Su	Cel	Mlb	Raf	St	Rbl	_D_Glu	Man	Glt	2-ket	Cit	NO_3_	‒V	37 °C		
*W. alni*	‒	‒	+	‒	‒	+	+	+	‒	‒	‒	v	+	+	‒	‒	+	+	‒	‒	+	‒
*W. anomalus*	v	‒	v	v	‒	‒	+	+	‒	+	+	v	+	+	‒	‒	+	+	+	v	+	‒
*W. arborarius*	+	l/‒	+	v	v	l/+	+	v	+	+	+	+	+	+	‒	n	+	+	n	‒	‒	‒
*W. bisporus*	‒	‒	+	w/‒	‒	+	+	+	‒	‒	‒	v	w/+	v	‒	‒	+	+	‒	‒	n	+
*W. bovis*	‒	‒	+	+	‒	v	+	+	‒	‒	v	‒	+	+	‒	‒	+	‒	‒	+	+	‒
*W. canadensis*	‒	‒	+	‒	‒	w/+	+	+	‒	‒	‒	v	+	w/+	‒	‒	+	v	‒	+	+	v
*W. chambardii*	+	‒	‒	‒	‒	‒	‒	+	‒	‒	‒	‒	‒	‒	‒	‒	w	‒	‒	‒	+	‒
*W. chaumierensis*	‒	‒	+	‒	n	‒	+	+	‒	‒	n	n	‒	‒	n	‒	n	‒	n	‒	n	‒
*W. ciferrii*	+	‒	w/+	w/+	‒	w/+	+	w/+	‒	+	+	+	+	+	‒	‒	+	+	+	w/‒	+	v
*W. edaphicus*	+	‒	+	w/‒	‒	+	+	+	+	+	+	+	+	+	l/‒	‒	+	+	+	w	n	‒
*W. hampshirensis*	‒	‒	+	‒	‒	s	+	+	‒	‒	‒	w/+	+	v	‒	‒	s	‒	‒	‒	+	‒
*W. kurtzmanii*	‒	‒	‒	‒	‒	‒	+	‒	w	‒	‒	‒	‒	w	‒	n	‒	+	‒	‒	‒	‒
*W. lynferdii*	+	‒	‒	‒	‒	‒	+	+	‒	+	‒	+	+	+	‒	‒	+	+	+	‒	+	‒
*W. menglaensis*	‒	‒	w	w	‒	w	‒	+	‒	‒	w	‒	+	+	‒	‒	+	+	+	n	‒	‒
*W. mori*	‒	+	‒	‒	w	‒	+	‒	‒	‒	‒	‒	n	+	‒	‒	w	‒	+	‒	‒	‒
*W. mucosus*	‒	+	+	‒	v	‒	+	+	‒	‒	+	‒	+	w/+	‒	+	‒	‒	‒	‒	+	‒
*W. myanmarensis*	+	‒	s	w	s	‒	+	s	‒	s	+	+	+	+	‒	n	+	+	+	+	+	‒
*W. ochangensis*	‒	‒	+	‒	‒	+	‒	+	‒	‒	‒	‒	+	+	‒	‒	‒	+	n	+	n	‒
*W. onychis*	‒	‒	+	v	v	‒	+	+	‒	+	‒	‒	+	+	‒	‒	+	‒	‒	+	+	‒
*W. orientalis*	+	n	w	w	w	w/‒	+	+	w	w	‒	w	n	w	n	n	‒	‒	‒	+	‒	+
*W. patagonicus*	w	‒	+	‒	‒	+	n	w	‒	w	w	‒	w	‒	‒	n	‒	‒	+	‒	n	‒
*W. pijperi*	‒	+	+	‒	‒	‒	‒	+	‒	‒	‒	‒	+	+	‒	‒	v	‒	‒	‒	+	‒
*W. psychrolipolyticus*	‒	‒	+	‒	+	+	+	+	‒	+	+	‒	+	+	n	n	+	+	n	‒	‒	‒
*W. queroliae*	‒	‒	+	+	‒	+	+	‒	‒	‒	‒	+	+	+	‒	‒	w/s	+	‒	+	‒	‒
*W. rabaulensis*	‒	‒	+	+	‒	v	+	+	‒	+	‒	+	+	+	‒	‒	+	‒	‒	+	+	‒
*W. scolytoplatypi*	+	‒	s	‒	‒	s	+	+	‒	‒	+	s	+	+	‒	‒	‒	+	‒	‒	+	‒
*W. siamensis*	s	‒	s	‒	‒	‒	+	w	‒	w	w	‒	w	w	‒	‒	‒	‒	‒	+	+	-
*W. silvicola*	+	v	+	+	‒	+	v	+	‒	‒	‒	+	+	v	‒	‒	v	+	‒	v	+	v
*W. spegazzinii*	+	‒	+	‒	‒	+	+	+	+	+	s	‒	+	+	‒	‒	w	+	+	+	+	‒
*W. strasburgensis*	+	‒	+	+	‒	+	+	+	‒	+	‒	+	+	+	‒	‒	+	‒	‒	v	+	‒
*W. subpelliculosus*	v	‒	v	v	v	‒	+	v	‒	+	v	v	+	+	‒	‒	+	+	‒	v	+	v
*W. sydowiorum*	+	‒	v	+	‒	+	+	+	+	+	v	+	+	+	‒	‒	+	+	+	‒	+	‒
*W. sylviae*	v	‒	s/‒	+	+	+,‒	w/‒	s/‒	‒	‒	w/+	s/‒	‒	‒	‒	‒	‒	v	+	+	‒	‒
*W. tratensis*	‒	‒	‒	‒	‒	‒	‒	v	‒	‒	‒	‒	v	v	‒	‒	‒	n	n	+	n	‒
*W. xylosicus*	‒	+	+	‒	‒	‒	+	+	‒	‒	‒	‒	‒	+	‒	w	‒	‒	n	‒	+	‒
*W. xylosivorus*	w/‒	‒	+	‒	‒	+	‒	+	‒	w	‒	‒	+	‒	n	‒	‒	+	+	n	‒	‒
*W. lannaensis*	‒	‒	+	‒	‒	+	‒	+	‒	‒	‒	‒	+	+	‒	‒	‒	+	w	‒	‒	‒
*W. nanensis*	+	‒	‒	‒	‒	‒	‒	+	‒	‒	‒	‒	‒	+	‒	‒	+	w	w	‒	‒	‒

* Ga = Galactose, Sor = L-Sorbose, _D_Xy = D-Xylose, _L_Ar = L-Arabinose, _D_Ar = D-Arabinose, Rh = L-Rhamnose, Su = Sucrose, Cel = Cellobiose, Mlb = Melibiose, Raf = Raffinose, St = Soluble starch, Rbl = Ribitol, _D_Glu = D-glucitol, Man = D-Mannitol, Glt = Galactitol, 2-ket = 2-ketogluconic acid, Cit = Citrate, NO_3_ = Potassium nitrate, ‒V = vitamin-free medium, 37 °C = Growth at 37 °C. “+” = strong growth or produce, “**‒**“ = absence of growth or not produce, “w” = weak growth, “v” = strain variable response, “l” = latent positive, “s” = slow positive, and “n” = no data.

## Data Availability

The DNA sequence data obtained from this study have been deposited in GenBank under accession numbers; D1/D2 domains (MT639220, MT623569, MT613722, MT613875 and MT623569) and ITS (OK135750, OK135752, OK135753, OK143510 and OK143511).
